# Identifying Opinion Leaders to Promote Organ Donation on Social Media: Network Study

**DOI:** 10.2196/jmir.7643

**Published:** 2018-01-09

**Authors:** Jingyuan Shi, Charles T Salmon

**Affiliations:** ^1^ Department of Communication Studies Hong Kong Baptist University Hong Kong China (Hong Kong); ^2^ Wee Kim Wee School of Communication and Information Nanyang Technological University Singapore Singapore

**Keywords:** social media, health promotion, organ donation, opinion leaders, social network analysis

## Abstract

**Background:**

In the recent years, social networking sites (SNSs, also called social media) have been adopted in organ donation campaigns, and recruiting opinion leaders for such campaigns has been found effective in promoting behavioral changes.

**Objective:**

The aim of this paper was to focus on the dissemination of organ donation tweets on Weibo, the Chinese equivalent of Twitter, and to examine the opinion leadership in the retweet network of popular organ donation messages using social network analysis. It also aimed to investigate how personal and social attributes contribute to a user’s opinion leadership on the topic of organ donation.

**Methods:**

All messages about organ donation posted on Weibo from January 1, 2015 to December 31, 2015 were extracted using Python Web crawler. A retweet network with 505,047 nodes and 545,312 edges of the popular messages (n=206) was constructed and analyzed. The local and global opinion leaderships were measured using network metrics, and the roles of personal attributes, professional knowledge, and social positions in obtaining the opinion leadership were examined using general linear model.

**Results:**

The findings revealed that personal attributes, professional knowledge, and social positions predicted individual’s local opinion leadership in the retweet network of popular organ donation messages. Alternatively, personal attributes and social positions, but not professional knowledge, were significantly associated with global opinion leadership.

**Conclusions:**

The findings of this study indicate that health campaign designers may recruit peer leaders in SNS organ donation promotions to facilitate information sharing among the target audience. Users who are unverified, active, well connected, and experienced with information and communications technology (ICT) will accelerate the sharing of organ donation messages in the global environment. Medical professionals such as organ transplant surgeons who can wield a great amount of influence on their direct connections could also effectively participate in promoting organ donation on social media.

## Introduction

### Organ Donation and Social Media

Since the 1960s, many countries have initiated organ donation programs, and at least eighty countries are now known to have a national organ donation program [1]. However, the organ donation program is still in its infancy in China. In China, the first nationwide organ donation program was not launched until 2013 [2]. This new opt-in organ donation program, however, has met with little success: approximately 1.5 million patients in China need an organ transplant each year, but only 10,000 of them are able to receive one [3]. By August 2017, only 310,620 Chinese people had registered as organ donors [4]. The great shortage of available organs in China underscores the need for organ donation promotion.

Social networking sites (SNSs, also called social media) are a popular platform for promoting organ donation in the United States [5,6] and in some other Western countries [7]. Organ donation campaigns using social media have yielded some promising results such as increasing organ donor consent rates. Weibo is one of the most popular social media platforms in China, with 600 million registered users [8] and is often regarded as the equivalent of Twitter in China. A previous content analysis revealed that people have shared their cultural beliefs, complex feelings, and concerns about organ donation, as well as their willingness to donate on Weibo [9]. Thus, Weibo is a potential platform for promoting organ donation in China. This paper focuses on the dissemination of organ donation tweets on Weibo and examines the opinion leadership in the retweet network of popular organ donation messages on Weibo using social network analysis.

To provide the background for the study, this paper will first review the literature on health communication campaigns and opinion leadership on social media. Following that literature review, this paper’s hypotheses are set forth. Finally, the Methods, Results, and Discussion are presented.

### Identifying Opinion Leadership for Social Media Health Campaigns

Recruiting opinion leaders has proven to be an effective strategy in Web-based organ donation promotions. For example, Stefanone et al [5] recruited peer leaders to endorse organ donation on social media and reported an increase in organ donor card requests and donor registration among college students at the end of the campaign. Such empirical evidence indicates that opinion leader interventions could encourage organ-donor registration behavior in SNS communities. In fact, opinion leaders have long been regarded as change agents in health campaigns because they are able to assist in the implementation of behavior change efforts: they can legitimize the behavior change program, convey the health messages, and act as role models for behavior change [10]. However, little research to date has identified opinion leaders for organ donation in the context of social media.

The classic two-step flow of communication hypothesis suggests that opinion leaders are individuals who directly receive information from mass media and, in turn, pass on what they know to their everyday associates through interpersonal communication [11,12]. Although this definition concerns opinion leaders’ access to information via mass media, subsequent definitions of opinion leaders have focused on the extent of influence, such as the impact on the opinions, attitudes, and behaviors that they exert on others [10,13,14]. Furthermore, recent research has discussed how the notion of opinion leadership on social media has evolved from earlier eras. Nowadays, many individual users—rather than merely a few opinion leaders— have substantial access to information, as well as the ability to share it with mass audiences in an instant [15]. It seems that opinion leaders’ privilege in accessing and disseminating information, which is suggested by the “two-step flow” hypothesis, no longer exists in the context of social media. Thus, opinion leadership on social media, may rest not only on the ability to access or disseminate information but also on the ability to bridge groups [14], build critical links in information dissemination [14], and trigger others in the network to share information [16].

Moreover, besides the “two step flow” process [12], other models of information dissemination, including direct one-step and complex network flows, also exist on social media [17,18]. A recent analysis of communication flows on Twitter reveals that general Twitter accounts receive information directly from traditional media and official accounts, as well as indirectly from intermediating amplifiers, who are individual or organizational users with public authority or public visibility [17]. The information dissemination on Twitter also follows a network step-flow that includes the coexistence of one-step flow, two-step flow, and a multi-step back-and-forth flow of communication among media and official accounts, general Twitter accounts, and amplifiers [17]. Thus, under such a complex model of information dissemination on social media, influential users emerge in local contexts, as well as in the overall communication network. In fact, such unique features of opinion leadership on social media are reflected in Bodendorf and Kaiser’s [19] recent conceptualization of online opinion leadership, which consists of two dimensions: local and global. Although both dimensions involve the ability of influencing and controlling information flow, they are slightly different from each other. Local opinion leadership refers to influence in a direct but limited environment; for example, a direct influence on one’s neighbors. Alternatively, global opinion leadership refers to indirect influence on others during the information exchange. For instance, this could be an ability to control the overall information flow in a whole network. In general, such local and global opinion leaders are crucial for the implementation of successful health promotions and interventions using social media platforms [20].

In terms of the operationalization of opinion leadership, scholars have employed various methods to identify opinion leaders who are able to assist in the implementation of behavior modification efforts [10]. Among all these means, the sociometric method has been regarded as “the most valid and reliable” method [10] and is “more precise than self-designating method” [21]. The sociometric method is able to capture not only direct flows of information but also a completed network of information dissemination and exchange. Within this network, several network metrics can be used to calculate the structural position a member has secured. Of all the network metrics, scholars have used indegree centrality most frequently to measure opinion leadership when employing the sociometric technique [20]. Previous sociometric studies have documented the positive relationships between indegree centrality and both self-reported and other-identified opinion leadership in offline and online environments [22-24]. In a retweet network on social media, indegree represents the number of direct ties a member receives from its neighbors, and members with a large number of indegree are prominent [25]. Hence, indegree centrality is a good indicator of one’s local opinion leadership [19]. However, this metric does not measure a member’s indirect connections in the network. To capture such indirect influence (ie, global opinion leadership), Bodendorf and Kaiser [19] proposed two other network metrics: closeness centrality and betweenness centrality.

Closeness is the sum of shortest distances from a member to all other members in the network [26], and the normalized version of closeness is divided into n – 1 (n is the number of all members in the network) [27]. In a directed network, this closeness can be calculated for sending (ie, out-closeness) and receiving (ie, in-closeness) [25]. In a retweet network of information on social media, a member with a high in-closeness score secures a position with a short distance to most others. It means that the tweet posted by this member will spread quickly to a random member in the network through network ties. Thus, we employ in-closeness instead of out-closeness as the indicator of opinion leadership for the retweet network in this study. Alternatively, betweenness measures how often a member falls along the shortest path between two other members in the network [26]. In a retweet network, members with high betweenness are usually acting as a bridge that connects different cluster of Weibo users. Members with high betweenness thus play gatekeeper roles in networks that control information flows and facilitate information dissemination beyond the boundaries of local groups [28,29]. Thus, this study adopts the Bodendorf and Kaiser [19] measures and employs three sociometric indicators for opinion leadership in a retweet network: indegree for local opinion leadership, and in-closeness and betweenness for global opinion leadership.

### The Predictors of Opinion Leadership

In additional to the indicators of opinion leadership, scholars have also long investigated the factors associated with opinion leadership. In 1957, Katz’s [11] classical article on opinion leaders proposed three predictors of personal influence: (1) personal attributes (who one is), (2) competence (what one knows), and (3) social position (whom one knows). This paper examines how these three factors are associated with opinion leadership for organ donation on a Chinese SNS. In this research, personal attributes include one’s activeness, verification status (ie, if the user is a verified account on Weibo), and geographic location; competence refers to one’s knowledge about medical issues in general; and social position refers to the numbers of followers and followings the user has on the SNS.

### Personal Attributes

#### Activeness

Sociability is the first factor related to leadership in offline and online contexts [30]. Individuals who engage in more communication activities can more easily obtain information and build relationships [31], thus having more potential to extend their reach to and influence others [32]. In the SNS context, research has found that one’s activeness on social media was positively associated with the number of retweets his or her posts received [33] and the probability of building communication ties with others [34]. Hence, the following hypothesis is proposed:

Hypothesis 1 (H1): a user’s activeness on an SNS is positively associated with his or her (1) local and (2) global opinion leadership for organ donation on social media.

#### Verification

On social media, some users’ accounts include a verified badge on their profile that shows the authenticity of their identities as key individuals or organizations. To obtain the verified badge, a verification request is usually submitted by the user and then confirmed by the SNS platform. Previous research has stated that the verification badge indicates a user’s credibility [33] or eliteness [35] on the SNS. For example, Zhang et al [33] claimed that verified accounts were perceived to be more credible than unverified accounts and found that messages posted via verified accounts attracted a larger number of retweets than did the messages posted via unverified accounts. Thus, having a verified badge on one’s profile on social media may lead to more influence on the SNS platform:

Hypothesis 2 (H2): compared with unverified users, verified users exhibit more (1) local or (2) global opinion leadership for organ donation on social media.

#### Information and Communication Technology Development in One’s Location

In addition to being associated with one’s activeness and identification on the SNS platform, opinion leadership in cyberspace may be subject to certain external environmental factors such as opportunities to access information and communications technologies (ICTs), including the Internet, cell phones, and personal digital assistants [36]. Opportunities for accessing and utilizing ICTs in one’s geographic location are closely tied to an individual’s ability to develop and maintain social relationships online [37,38]. Indeed, Lyons and Henderson [39] revealed that computer skill and Internet self-efficacy are positively associated with a person’s opinion leadership in a computer-mediated environment. Moreover, a recent study on users’ influence in SNS communities found that, within a Weibo community about human immunodeficiency virus or acquired immune deficiency syndrome, users coming from areas with well-developed ICTs secured more influential positions in their follower-following network than did those who came from areas with underdeveloped ICTs [36]. Thus, it is reasonable to expect a positive effect of the level of ICT development in one’s location on his or her influence on the organ donation topic in virtual communities:

Hypothesis 3 (H3): the degree of ICT development in a user’s location is positively associated with his or her (1) local and (2) global opinion leadership for organ donation on social media.

### Competence

An individual’s expertise or knowledge about a social issue has long been regarded as a critical contributor to his or her stature as an opinion leader on the topic [11,32]. The research on product diffusion has found that opinion leaders are more knowledgeable about the product than nonleaders and that individuals who are superior in professional knowledge are also more likely to become opinion leaders in computer-mediated environments as well [39]. In terms of organ donation, medical knowledge is critical to improving the willingness of donation and reducing refusal from potential donors’ relatives [40]. On Weibo, physicians are a special group of users. Some of them not only received verification from the platform but also had detailed profile information that showed their professional position in a clinic, hospital, or university. Thus, this group of users who possess medical knowledge are considered credible and authoritative and exhibit more opinion leadership for organ donation:

Hypothesis 4 (H4): users with a medical-focused profile on social media exhibit more (1) local and (2) global opinion leadership for organ donation than others without medical-focused profiles.

### Social Positions

SNS users not only integrate their offline social relationships into a cyber network but also develop new online social ties on social media [15]. In a network of social relationships on social media, a user disseminates information directly to his or her followers. Meanwhile, the user himself or herself is exposed to the information his or her connections send out. Having a large number of followers (those who followed the focal user) enables a user to disseminate information efficiently, whereas a large number of followings (those whom followed by the focal user) provides the user with a broad source of information. Indeed, previous research has shown that individuals who are well connected on social media are more influential than others in the virtual environment [18]. For example, Zhang et al [33] found that the number of followers a person had was positively associated with the number of retweets and comments that person’s posts received. Thus, users with numerous followers and followings obtain a well-connected location and are influential in the SNS network:

Hypothesis 5 (H5): the number of followers a user has is positively associated with his or her (1) local and (2) global opinion leadership for organ donation topic on social media.

Hypothesis 6 (H6): the number of followings a user has is positively associated with his or her (1) local and (2) global opinion leadership for organ donation on social media.

## Methods

### Data Collection and Sample

Weibo is a Twitter-like microblogging service site that was launched in 2009 and that has become one of the most popular social media platform in China, with 600 million registered users [8]. This study used the built-in Weibo main search function to retrieve all messages about organ donation posted from January 1, 2015 to December 31, 2015. The search keywords included “donation/donating/donated” and the names of organs and tissues listed on the Chinese organ donor registration application (including organ/organs, body/bodies, kidney/kidneys, liver/livers, heart/hearts, lung/lungs, pancreas, small intestines, and cornea). Python Web crawler was employed to extract all available searching results on January 10, 2016, which consisted of 7465 Weibo posts. To eliminate irrelevant messages, the Weibo posts were manually coded into two categories: (1) relevant, which discusses organ donors, organ recipients, organ donation systems, or organ donation policy and ethics and (2) irrelevant, which discusses issues not relevant to the aforementioned organ donation topics. Two native Chinese speaker coders independently coded 10.04% (750/7465) of the posts, randomly chosen from the data, and the intercoder reliability Cohen kappa [41] was .92. Next, all the 7465 posts were split in half and separately coded by the two coders, and 6701 messages (89.77%, 6701/7465) were coded as organ donation messages.

The popular organ donation messages were defined as the messages whose number of retweets ranked in the top three percentiles out of all 6701 messages (n=206). The retweet network of the 206 popular messages was extracted using Python Web crawler in April 2016, resulting in a retweet network with 505,047 unique Weibo users. Next, Python Web crawler was employed to extract the profile information of the Weibo users who received at least one retweet from others in the retweet network (n=44,074). The Python Web crawler extracted all existing accounts as of April 2016, which included 43,510 users. The information about these users’ profiles included the account’s username, verification status, self-introduction, self-reported location and gender, as well as his or her number of followers, followings, and posts on Weibo.

### Constructing the Retweet Network

The retweet network of the popular organ donation messages was constructed such that if user *i* retweets the post of member *j*, then *i* was connected to *j*. The direction of the tie was from *i* to *j*, with a weight that equals the number of times that *i* retweets posts from *j*. Thus, the retweet network is a directed, weighted network. We constructed and analyzed the retweet networks using the igraph package [42] in R.

### Measures and Analytical Design

#### Opinion Leadership

An individual’s opinion leadership was measured via three network metrics from the retweet network, including his or her indegree for local opinion leadership, as well as in-closeness and betweenness for global opinion leadership. The indegree ranged from 1 to 59,061 with a mean of 12.51 (standard deviation [SD] 515.32). The in-closeness ranged from 3.92e-12 to 6.20e-12 with a mean of 3.95e-12 (SD 1.17e-13). The betweenness ranged from 0 to 58,442,335.20 with a mean of 80,485.50 (SD 939,107.11).

#### Activeness

The measurement of a user’s activeness on Weibo was adapted from Zhang et al [33], which used the total number of messages a user has posted. Among the 43,510 users, the number of posts ranged from 0 to 677,495 with a mean of 11,170.01 (SD 16,617.82).

#### Verification

If a user had a verified badge in his or her Weibo profile, this account was regarded as a verified account. It was a dichotomous variable: *verified*=1 (4158/43,510, 9.5%) and *unverified*=0 (39,352/43,510, 90.4%).

**Table 1:** The correlation matrix among all continuous variables.

Variable	1	2	3	4	5	6	7
1. Indegree	_						
2. In-closeness	0.266^a^	_					
3. Betweenness	0.117^a^	0.567^a^	_				
4. The number of followers	0.587^a^	0.163^a^	0.043^a^	_			
5. The number of followings	0.068^a^	0.115^a^	0.076^a^	0.133^a^	_		
6. Activeness on Weibo	0.117^a^	0.163^a^	0.200^a^	0.171^a^	0.361^a^	_	
7. Location	0.037^a^	0.043^a^	0.041^a^	0.045^a^	0.027^a^	0.111^a^	_

^a^*P*<.01.

#### Information and Communication Technology Development

The user’s location was recoded as a continuous variable according to its degree of ICT development. According to China’s ICT development index [43] and the digital access index for all countries in the world [44], the location was coded into a continuous variable from 1 to 6, with a higher value representing a better degree of ICT development (mean=3.83, SD=1.29).

#### Medical-Focused

If a user has a medical-focused profile which lists the user’s professional position in a clinic, hospital, or university, this variable was coded as 1 (n=84).

#### The Numbers of Followers and Followings

This information was listed in users’ profiles. The number of followers of all the users ranged from 0 to 50,563,948 with a mean of 38,801.45 (SD 827,423.30). The number of followings ranged from 1 to 5832 with a mean of 543.84 (SD 545.83).

#### Analytical Design

The general linear model (GLM) was employed for analysis. Due to highly skewed distributions, indegree, in-closeness, betweenness, activeness, and the numbers of followers and followings were square root transformed to meet the assumptions of GLM. The correlation matrix among continuous variables is presented in Table 1.

## Results

### Mapping the Retweet Network of Popular Organ Donation Messages

**Figure 1:**
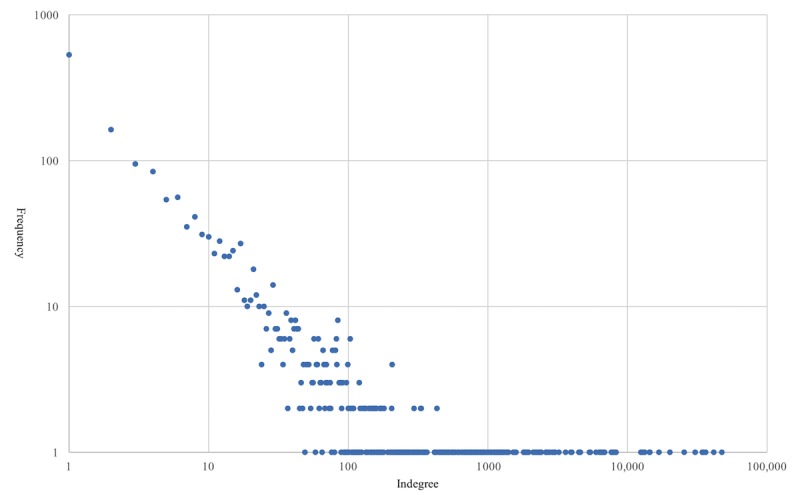
The log transformed distribution of indegree.

**Figure 2:**
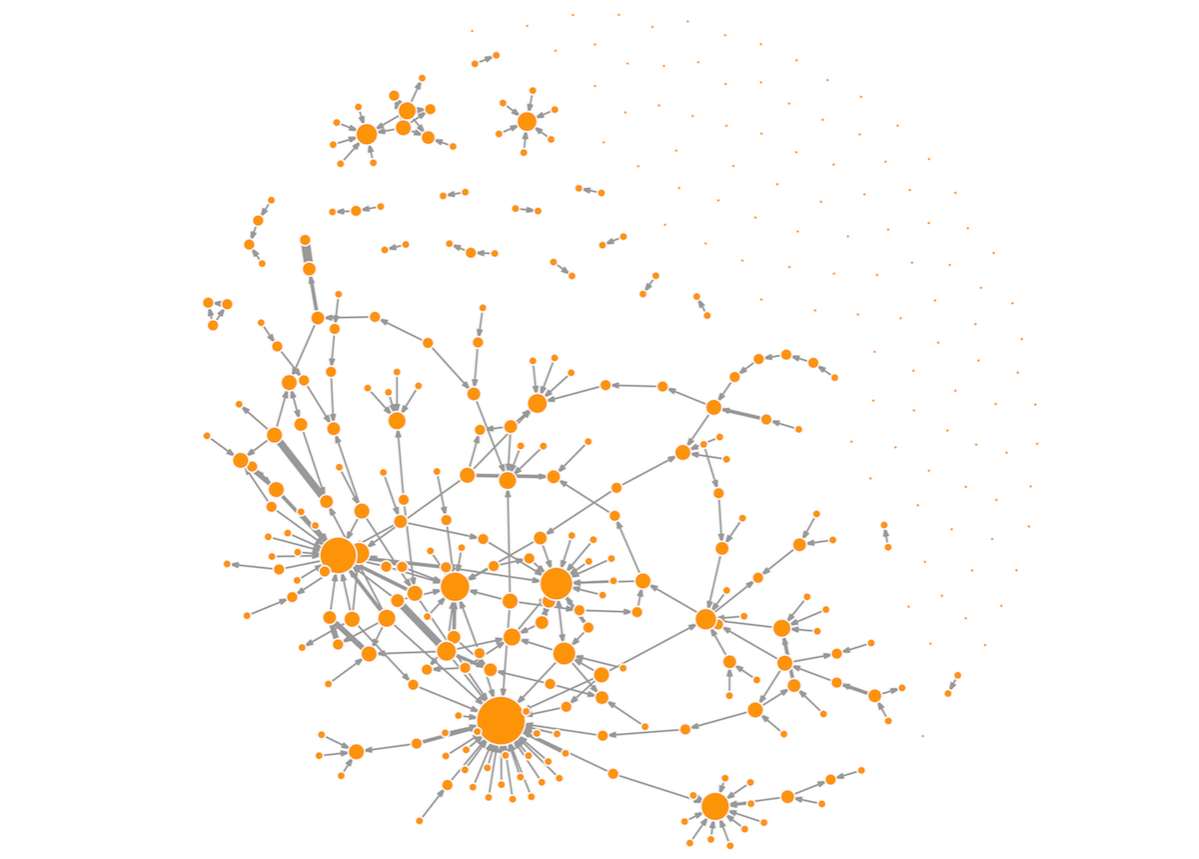
Visualization of the sharing network of popular organ donation messages. The figure includes only nodes whose indegree equal or larger than 50. Nodes represent Weibo users (n=362). The size of node depends on its indegree. The larger the node, the greater amount of retweets the user received. Lines represent retweet relationship between Weibo users. The weight of line depends on the number of retweets.

The retweet network is a connected network including 505,047 nodes and 545,312 edges. The length of the maximum distance between nodes (ie, diameter) in this network is 21. The network has a low density: only 0.0002% of possible edges between all the nodes are connected. The reciprocity values and clustering coefficient of this network are extremely low at 0.0016 and 0.000003, respectively. The low values of density, reciprocity, and clustering coefficient indicate a sparse network. In addition, indegree centralization is 0.1169, and outdegree centralization is 0.00017 for this network. This indicates that, in terms of indegree, links are retweeted disproportionately to a small group of users. The distribution of indegree within this retweet network is highly skewed (see Figure 1). All the network-level statistics indicate that this retweeting network is a sparse and centralized network. Table 2 summarizes the network-level statistics. Figure 2 visualizes this retweet network with nodes whose indegree is equal or larger than 50.

**Table 2:** The network-level characteristics of the retweeting network.

Social network metric	Definition	Possible range	Value
Size	The number of nodes (eg, users) in the network	N/A^a^	505,047
Diameter	The largest geodesic distance, which is the shortest distance from one node to another in the network	N/A	21
Density	The proportion of all possible dyadic connections that are presented in the network	0-1	0.000002
Reciprocity	The proportion of all pairs in the network that have a reciprocated tie between them	0-1	0.0016
Clustering coefficient	The degree to which nodes in the network tend to cluster together	0-1	0.000003
Indegree centralization	The extent to which the distribution of indegree centrality in the network deviates from a perfectly equal distribution	0-1	0.1169
Outdegree centralization	The extent to what the distribution of outdegree centrality in the network deviates from a perfectly equal distribution	0-1	0.00017

^a^N/A: not applicable.

**Table 3:** The GLM effects of predictors on individuals’ opinion leadership.

Predictors		Local opinion leadership		Global opinion leadership			
		Indegree		In-closeness		Betweenness	
		Coefficients	Standard error	Coefficients	Standard error	Coefficients	Standard error
Intercept		1.57^a^	0.31	0.000002^a^	0.000000003	−53.76	29.67
**Personal attributes**							
	Activeness on Weibo	0.001^a^	0.0003	0.00000000006^a^	0.000000000003	0.89^a^	0.03
	Verification	−0.33^a^	0.05	−0.0000000007	0.0000000005	−17.36^a^	4.98
	Location	0.02	0.01	0.0000000005^a^	0.0000000001	3.72^a^	1.05
**Competence**							
	Medical-focused	1.14^a^	0.30	0.000000001	0.000000003	−8.02	29.35
**Social positions**							
	The number of followers	0.01^a^	0.00008	0.00000000002^a^	0.0000000000008	0.02^b^	0.01
	The number of followings	−0.003	0.002	0.0000000002^a^	0.00000000002	0.34^c^	0.16
	∆ *R*^2^	34.4%		5.2%		4.2%	
**Control variable**							
	Gender (female=1, male=0)	−0.14^a^	0.03	−0.000000002^a^	0.0000000003	7.60^c^	3.16
	∆ *R*^2^	0.0%		0.0%		0.0%	
	Total *R*^2^	34.4%		5.2%		4.2%	

^a^*P*<.001.

^b^*P* ≤.01.

^c^*P*<.05.

### The Predictors of Opinion Leadership

The GLM results are reported in Table 3. The results showed that all predictors accounted for 34.4% of the variance in indegree, 5.2% of the variance in in-closeness, and 4.2% of the variance in betweenness. Personal attributes, professional knowledge, and network positions significantly affected the number retweets one node received (ie, local opinion leadership). Nevertheless, in terms of global opinion leadership, only personal attributes and network positions were significant predictors. Professional knowledge did not significantly affect this type of opinion leadership.

#### Personal Attributes

H1 predicted a positive effect of users’ activeness on Weibo on (1) local and (2) global opinion leadership on the organ donation topic. The analysis revealed that the number of messages one posted on Weibo was significantly and positively associated with one’s local opinion leadership, *B*=.001, *P*<.001, as well as global opinion leadership: *B*=6e-11, *P*<.001 for in-closeness and *B*=.89, *P*<.001 for betweenness. Hence, the data were consistent with H1 (1) and H1 (2).

H2 proposed that, compared with an unverified user, a verified user exhibits more (1) local and (2) global opinion leadership within the retweet network about organ donation. However, the results showed an opposite direction of effect. The unverified users exhibited significantly more local opinion leadership than verified users within the retweet network, *B*=−.33, *P*<.001. With regard to the global opinion leadership, they obtained a significantly higher value of betweenness than verified users, *B*=−17.36, *P*<.001. The results of the other indicator, in-closeness, did not reach significance. Hence, the unverified users showed significantly more opinion leadership than did verified users, and the data were inconsistent with H2 (1) and H2 (2).

H3 predicted positive effects of ICT development level in users’ location on his or her (1) local and (2) global opinion leadership in the retweet network of organ donation message. The results showed that level of ICT development was not significantly associated with local opinion leadership. However, it was positively associated with two global opinion leadership indicators: in-closeness, *B*=5e-10, *P*<.001 and betweenness, *B*=3.72, *P*<.001. Thus, the data were inconsistent with H3 (1) but consistent with H3 (2).

#### Competence

H4 made predictions about the effects of professional, medical knowledge on (1) local and (2) global opinion leadership in the retweet network of organ donation messages. The results showed that medical-focused users significantly exhibited more local opinion leadership than other users, *B*=1.14, *P*<.001. However, such effect on global opinion leadership did not reach significance. Thus, the data were consistent with H4 (1) but inconsistent with H4 (2).

#### Social Positions

H5 and H6 considered the effects of social position on Weibo on one’s opinion leadership on the organ donation topic. H5 predicted a positive effect of the number of followers on (1) local and (2) global opinion leadership. The results showed that users with a higher number of followers were more likely to exhibit more local as well as global opinion leadership in retweet network about organ donation: *B*=.01, *P*<.001 for indegree, *B*=2e-11, *P*<.001 for in-closeness, and *B*=.02, *P*=.01 for betweenness. Hence, the data were consistent with H5 (1) as well as H5 (2). H6 anticipated a positive effect of the number of followings on (1) local and (2) global opinion leadership. The analysis revealed that users with a higher number of followings showed more global opinion leadership: *B*=2e-10, *P*<.001 for in-closeness and *B*=.34, *P*=.03 for betweenness. However, it did not significantly affect local opinion leadership. Therefore, the data were inconsistent with H6 (1) but consistent with H6 (2).

## Discussion

### Major Findings and Implications

This study investigates organ donation information on Weibo by mapping its sharing (ie, retweet) network and examining the local as well as global opinion leadership in the network. This work explores the role of personal attributes, professional knowledge, and social position in obtaining influence according to Katz’s [11] treatise. The findings reveal that all three factors predict individuals’ local opinion leadership in the retweet network. Alternatively, personal attributes and social position, but not professional knowledge, are significantly associated with global opinion leadership. This study’s findings significantly improve the understanding of organ donation information on social media and will be instrumental in the design of organ donation promotions on social media.

The sharing network of popular organ donation messages on Weibo is extremely sparse and centralized, resembling a star-like network structure. Only a very small portion of users in this network receives retweets from others, whereas more than 90% of users do not receive any retweets from others and occupy peripheral positions in the network. This result indicates that few central users control the flow of organ donation information and could act as critical peer leaders in organ donation promotions on Weibo. After mapping the network, subsequent analysis explores how individual and social factors affect these users’ ability to influence the information flow (ie, opinion leadership). In addition, the opinion leadership on social media is conceptualized as a two-dimensional construct, including a direct influence in neighborhood (ie, local opinion leadership), as well as an indirect impact in the whole environment (ie, global opinion leadership).

The findings show that two personal attributes are significant predictors of both local and global opinion leadership on organ donation: activeness on Weibo and verification status. In detail, compared with inactive users, active users are more likely to show greater local and global opinion leadership in the organ donation information diffusion network on Weibo. This finding is consistent with theories on developing influence [30,32], as well as previous empirical research on Weibo [33,34]. Hence, the activeness of users could be a direct and simple criterion of selecting peer leaders for SNS organ donation promotions. These users exert strong influence on their neighbors and spread the information throughout the entire network and target audience.

Nevertheless, the other personal attribute, verification status, negatively impacts opinion leadership, which is the opposite of H2’s prediction. This study found that, compared with verified users, unverified users are more likely to show greater local as well as global opinion leadership about organ donation on Weibo. One possible explanation is that a user’s influence on social media is topic-sensitive [45]. Although a previous study claimed that verified users are perceived to be more credible than unverified ones and that their posts on Weibo received more retweets than others’ posts, this study was not topic-specific and contained nine different topics from personal interests to political news [33]. The current study, however, focuses on a specific topic—organ donation. It is possible that the role of verification status varies for different topics and that Weibo users would turn to unverified rather than verified users for opinions about organ donation.

The other possible explanation could be that, in general, verified users may enjoy less rather than more credibility than unverified ones. Indeed, the Chinese government has hired a large number of people to fabricate posts on popular websites and social media, and the number of pseudonymous and deceptive social media posts could reach 488 million a year [46]. Although the government has never publicly or officially admitted such an operation, the general public in China is fully aware of it. Thus, the verification badge on Weibo could backfire. People may regard users who have received official verification as government employees as individuals who may intentionally manipulate public opinion on Weibo. This could be why unverified users exhibit greater local and global opinion leadership about organ donation than verified users on Weibo. However, both explanations need further examination. Health communication professionals should be aware of this counterintuitive finding and be more cautious when choosing verified users on Weibo as peer leaders to promote organ donation.

The third personal attribute examined in this study is the level of ICT development in one’s location. Unlike the abovementioned two attributes, which are relevant to a user’s activities and identity on Weibo, this one is an environmental factor. The results show that a person’s direct impact on the neighborhood (ie, local opinion leadership) is highly associated with his or her characteristics and identity on Weibo but not with the ICT development level in his or her area. A user’s indirect influence on other users (ie, global opinion leadership) depends on that user’s characteristics on Weibo as well as this environmental factor. Indeed, a previous study found that ICT development was highly associated with users’ influence in friendship networks on social media [36]. This study extends the previous research on social (ie, following-follower) networks to an information network on Weibo. The results show that, with regard to the organ donation topic on Weibo, although the ICT development level in one’s local network does not affect his or her direct influence on others, it significantly impacts the user’s indirect influence and the ability to control information flows in the whole environment. Therefore, users from ICT mature areas can be recruited as peer leaders for SNS organ donation promotion targeting on a wide range of audience groups.

The second type of opinion leadership predictor suggested by Katz [11] is professional knowledge on the topic. This study shows that users with medical knowledge exhibit significantly greater local opinion leadership about organ donation on Weibo than users without such knowledge. However, this effect is not significant for global opinion leadership. It is possible that a user’s professional knowledge on a certain topic is critical for obtaining opinion leadership when the information flows follow the “two-step flow.” Hence, as the results reveal, it significantly affects local opinion leadership on the organ donation topic. However, competence or credibility may become less important when individuals want to wield global influence over the retweet network, within which multiple avenues of information flow coexist. Thus, although medical professionals are influential organ donation opinion leaders, their impact is limited to their close neighbors.

The last predictor of opinion leadership included in this study is a user’s social position on Weibo. The results reveal that compared with obtaining local opinion leadership, securing global influence requires a well-connected social location in the network. For local opinion leadership, the number of followers, but not followings, is a significant predictor. The number of a user’s followers is the number of users on Weibo who will be directly exposed to his or her posts (ie, the user’s direct audience). As documented in these results, the larger the size of a user’s direct audience, the greater level of that user’s local influence. On the other hand, global opinion leadership depends on not only the size of one’s direct audience but also the size of the user’s information sources (ie, followings) on Weibo. Users with large audiences and many information sources occupy well-connected positions in the network and have updated information on the topic, thus exhibiting more global opinion leadership than others who occupy peripheral social positions. For public health professionals, they may recruit peer leaders according to campaign objectives. A user with a large direct audience will be competent to impact his or her neighbors, but only users with a large audience and many information sources will be capable of controlling the dissemination of organ donation information on Weibo.

Although previous organ donation campaigns have employed social media, the campaign advertisements and strategies were specifically designed for college students, and the information dissemination was mainly controlled by the researchers [5,6]. This procedure, however, may have limited generalizability to other populations and also to other contexts outside the confines of a controlled environment [47]. To explore opinion leadership on social media in a natural context, this study analyzes the general public’s organ donation discussion and retweeting behavior on a popular Chinese social networking site. This unobtrusive approach offers implications for recruiting peer leaders on social media to promote organ donation. In sum, the findings of this study indicate that health campaign designers may recruit peer leaders in SNS organ donation promotions to facilitate information sharing among target audience. Users who are unverified, active, well connected, and experienced with ICT will accelerate the sharing of organ donation messages in the global environment. Medical professionals such as organ transplant surgeons who can wield a great amount of influence on their direct connections could also effectively participate in promoting organ donation on social media.

### Limitations and Future Research

There are several areas worthy of further research in opinion leadership in the topic of organ donation on social media. First, examining the retweet paths of all the organ donation messages (n=6701) would yield an extremely large dataset and be computationally intensive, so this study focuses on only the most popular messages. In fact, this study initiates an exploration of opinion leadership of organ donation promotion on social media with an innovative and advanced method. Future research may replicate this research on other SNS platforms or with a larger dataset. Second, this study analyzes a snapshot of the retweet network instead of a dynamic diffusion network that evolves over time. Subsequent work may employ more sophisticated data mining and data analyzing techniques to detect how organ donation messages go viral on social media and who facilitates the dissemination, which would offer valuable information for future SNS organ donation promotion. Third, apart from medical knowledge, some other factors such as experience with organ donation may contribute to a user’s opinion leadership on the topic of organ donation. Future studies could explore other measures or indicators of users’ competence on the topic of organ donation. Fourth, this study examines the retweet network of all organ donation tweets regardless of their content. However, the structure of the retweet network may vary by how organ donation is covered or framed in the tweets. Future research should investigate whether content shapes the retweet paths and opinion leadership. For example, are myths about organ donation disseminated the same way as stories about an organ recipient? Findings from this research will greatly enhance the design and implementation of organ donation campaigns using social media.

## Abbreviations

GLM: general linear model
ICT: information and communications technology
SD: standard deviation
SNS: social networking site
